# Surveillance of bacteria *Pseudomonas aeruginosa* and MRSA associated with chronic suppurative otitis media^☆^

**DOI:** 10.1016/j.bjorl.2016.03.008

**Published:** 2016-04-22

**Authors:** Sibanarayan Rath, Saumya Ranjan Das, Rabindra Nath Padhy

**Affiliations:** a Siksha ‘O’ Anusandhan University, Institute of Medical Sciences & Sum Hospital, Central Research Laboratory, Kalinga Nagar, Odisha, India; b Siksha ‘O’ Anusandhan University, Institute of Medical Sciences & Sum Hospital, Department of ENT, Kalinga Nagar, Odisha, India

**Keywords:** Chronic suppurative otitis media, *Pseudomonas aeruginosa*, MRSA, Intracranial complications, Otite média crônica supurativa, *Pseudomonas aeruginosa*, MRSA, Complicações intracranianas

## Abstract

**Introduction:**

Suppurative otitis media is a critical disease causing perforation of the tympanic membrane associated with changes of the mucoperiosteum of the middle ear cleft.

**Objective:**

To isolate causative bacteria from chronic suppurative ear discharges and to ascertain their antibiotic profiles, of patients attending outpatients department in 3 years.

**Methods:**

For isolation of bacteria, samples of ear discharges were grown in suitable media and bacteria were subjected to antibiotic profiling by the Kirby–Bauer's method with presently used antibiotics.

**Results:**

A total of 1043 bacteria were isolated, including *Pseudomonas aeruginosa* and methicillin resistant *Staphylococcus aureus*, along with 121 fungal isolates. Among 371 *P. aeruginosa* isolates, tobramycin 30 had the highest susceptibility rate 93.2%, followed by ceftazidime 30, 91.5% and amikacin 10 μg/disk 64.4%. Of 359 *S. aureus* isolates, there were 236 coagulase negative *S. aureus* + methicillin sensitive *S. aureus* isolates, while 123 isolates were methicillin resistant *Staphylococcus aureus* with 95.2% isolates susceptible to cloxacillin 15, 83.3% isolates to erythromycin 15 and 78.5% isolates to gentamicin 30 μg/disk. Of 1164, 49 patients presented post aural abscess, 12 patients had intracranial complications, 9 patients had facial palsy and 3 patients had labyrinthitis. More than 90% *P. aeruginosa* and 90% *S. aureus* isolates were sensitive to tobramycin 30 and cloxacillin 30 μg/disk, respectively.

**Conclusion:**

Multidrug resistant strains of *P. aeruginosa* were more prevalent than those of *S. aureus* in ear discharges. Tobramycin and cloxacillin may be included in the formulatory antibiotic regimen to overcome bacterial infections in chronic suppurative otitis media.

## Resumo

**Introdução:**

Otite média supurativa é uma doença importante que causa perfuração da membrana timpânica além de alterações do mucoperiósteo da orelha média.

**Objetivo:**

Isolar as bactérias causadoras a partir da secreção auricular crônica e verificar seus perfis de sensibilidade aos antibióticos em pacientes ambulatoriais durante três anos.

**Método:**

Para o isolamento das bactérias, as amostras de secreções auriculares foram cultivadas em meios adequados e as bactérias foram submetidas à detecção de perfis de sensibilidade aos antibióticos usando o método de Kirby-Bauer para antibióticos usados na atualidade.

**Resultados:**

No total, 1.043 bactérias, incluindo *Pseudomonas aeruginosa* resistentes à meticilina e *Staphylococcus aureus*, e 121 fungos isolados foram identificados. Entre 371 isolados de *P. aeruginosa*, tobramicina 30 μg/disco apresentou a maior taxa de susceptibilidade (93,2%), seguida por ceftazidima 30 μg/disco (91,5%) e amicacina 10 μg/disco (64,4%). De 359 isolados de *S. aureus*, 236 eram *S. aureus* coagulase-negativos + *S. aureus* sensíveis à meticilina (MSSA), enquanto 123 eram MRSA com 95,2% de suscetibilidade à cloxacilina 15 μg/disco, 83,3% sensíveis à eritromicina 15 μg/disco e 78,5% à gentamicina 30 μg/disco. Entre 1.164 pacientes, 49 apresentaram abscesso aural, 12 apresentaram complicações intracranianas, nove apresentaram paralisia facial e três apresentaram labirintite. Mais de 90% das *P. aeruginosa* isoladas e de 90% de *S. aureus* eram sensíveis à tobramicina 30 μg/disco e cloxacilina 30 μg/disco, respectivamente.

**Conclusão:**

Cepas multirresistentes de *P. aeruginosa* foram mais prevalentes que as de *S. aureus* nas secreções auriculares. Tobramicina e cloxacilina podem ser consideradas na formulação de regime de antibióticos para tratar as infecções bacterianas na OMCS.

## Introduction

The generic term, ‘otitis media’ includes widely, cases of ‘acute otitis media’ (AOM) and cases of ‘otitis media with effusion’ (OME); basically these are non-suppurative. Moreover, ‘chronic otitis media’ (COM) is the gathering of pus from suppurations when infections are chronic; eventually, chronic suppurative otitis media (CSOM) are with inflammation and the production of pus.^1^ Additionally, CSOM may remain inactive with the potential to be active occasionally, leading to a perforation of the tympanic membrane associated with changes of the mucoperiosteum of the middle ear cleft with/without mucoid or mucopurulent otorrhea.^1^, ^2^, ^3^ It takes usually 2 or 3 weeks or more duration, for the disease to be recognized as active. A healed COM may have permanent abnormalities of the pars tensa; but with an intact pars tensa the occurrence of COM is rare.^3^ CSOM could lead to hearing loss, intermittent otalgia causing psychological trauma. In CSOM, the most causative bacteria are *Klebsiella* sp., *Proteus* sp., *Pseudomonas aeruginosa* and *Staphylococcus aureus*.^4^ And other bacteria commonly isolated from patients with AOM are *Haemophilus influenzae*, *Moraxella catarrhalis* and *Streptococcus pneumonia*.^5^ Moreover, *P. aeruginosa* had been seen as a notorious pathogen in this hospital too.^6^ Mainly found in wounds and urinary tract, it finds ways as bloodstream infection (BSI) to innards causing comorbidities.^7^ Indeed, the ability of these organisms to form biofilm that may contribute to their frequency in CSOM.^8^ As it is known, the rate of invasion of a pathogenic bacterium directly depends on its level of drug resistance, apart from immune-conditions of patients.^9^

Particularly, several clonal variants of *S. aureus* were resistant to the penicillin group of antibiotics, after which methicillin/oxacillin were introduced for the control. Subsequently, methicillin resistant *S. aureus* (MRSA), causing surgical site infections and wound emerged.^10^ The most gruesome situation is that MRSA strains have emerged with concomitant/subsequent resistance to most commonly used antibiotics of groups, aminoglycosides, macrolides, fluoroquinolones, chloramphenicol and tetracycline and many more such as, to cephalosporins, cefems and other β-lactams, ampicillin-sulbactam, amoxicillin-clavulanic acid, ticarcillin-clavulanic acid, piperacillin-tazobactam and the carbapenem, imipenem. Thus, MRSA isolates are MDR too.^11^ Moreover, the most dominating fungal species were of *Candida* and *Aspergillus* along with MRSA; and in a surveillance, 50% patients were diagnosed with candidiasis.^10^ Indeed, *Candida albicans* was originally a harmless fungus in healthy persons, but its causes superficial to life-threatening uncontrollable systemic infections due to the emergence of antifungal resistance.^12^

This work describes surveillance of bacterial flora from ear discharges of patients attending the Outpatients Department (OPD) of ENT department of the hospital, in the last 3 years. And the cited two fungi were too isolated along with bacteria. Antibiograms of isolated bacterial taxa were determined to assess the spectrum of CSOM that would help in rescheduling antimicrobial stewardship program of the hospital or the zone of central Odisha.

## Methods

A total of 1230 pus discharges from clinically diagnosed CSOM cases were collected, during January 2012 to January 2015 with sterile cotton swab sticks. Pus swabs were cultured on blood and MacConkey agar plates that were incubated at 37 °C overnight for pathogenic bacteria, which were identified according to the standard method used for bacteria and concomitantly for fungi.^6^, ^13^, ^14^ Antibiotic susceptibility tests of isolated bacteria were done according to Clinical Laboratory Standard Institute guidelines, as described.^15^, ^16^ Standard antimicrobial disks (HiMedia, Mumbai) used for *S. aureus* were, oxacillin, cotrimoxazole, penicillin, cloxacillin, gentamicin, chloramphenicol, ciprofloxacin and vancomycin; similar disks used for *P. aeruginosa* were gentamicin, chloramphenicol, ciprofloxacin, ceftazidime, piperacillin, carbenecillin and tobramycin.

### Antibiotic sensitivity and detection of MRSA

The standard MTCC number 7443 strain and all the isolated *S. aureus* strains were subjected to antibiotic sensitivity tests with antibiotics, by the Kirby-Bauer's method (disk diffusion) detailed previously.^11^ For the detection of MRSA, chromogenic agar media test was used; pure clinical isolates of *S. aureus* were streaked onto MRSA-agar media as described.^11^ Muller-Hinton Agar (MHA) plates were incubated at 37 °C for 18 h and inhibition-zone diameters were measured. A value of inhibition-zone diameter less than 22 mm was reported as oxacillin resistant and that more than 21 mm was considered as oxacillin sensitive.^11^

### Identification of fungi

Direct microscopic examination of the cotton swab with samples was carried out by mounting sample lots treated with 1–2 drops of 10–20% KOH for 15–30 min. Each specimen-lot was inoculated on two sets of Sabouraud's dextrose agar slopes, one set with chloramphenicol and the other set with cycloheximide (chloramphenicol – 0.05 mg/mL, cycloheximide – 0.5 mg/mL). Cultures were incubated at room temperature for 4–6 weeks and were observed regularly for possible growth. Fungal isolates were identified on the basis of duration of growth and surface morphology of colonies, as well as pigment production on the reverse and microscopic examination of hyphae in lacto phenol cotton blue preparation.^6^, ^16^

## Results

From 1230 collected samples, 1164 bacterial and fungal colonies grew as 629 single and 535 mixed colonies on agar plates and no microbial growth was seen with 66 samples. There were 1043 bacterial and 121 fungal isolates in total. The most common causal bacteria isolated were 220 isolates of *S. aureus* with and 188 isolates of *P. aeruginosa*; and 19 isolates of *S. aureus* were MRSA, and 64 isolates were coagulase negative *S. aureus* (CONS). Bacteria, *P. aeruginosa* was isolated in 183 of the total 1164 samples that yielded mixed colonies of *S. aureus*, *Klebsiella* sp. and *Proteus* sp., followed by *Escherichia coli*, given in Table 1. Fungi accounted for 63 isolates of *Aspergillus* sp. and 68 isolates of *Candida* sp. as both single and mixed colonies from 1164 growth-yielding samples, given in Table 1.

**Table 1:** Growth of bacteria and fungi in cultures of ear discharge samples of OPD patients with CSOM as single colony and mixed colonies.

Organisms	Single colony isolates *n*_1_ = 629 (100)	Mixed colony isolates *n*_2_ = 535 (100)	Total isolates (*n*_1_ + *n*_2_) = *n* = 1164 (100)
*Enterobacter* sp.	19 (03.0)	–	19 (01.6)
CONS	64 (10.1)	–	64 (05.4)
MRSA	19 (03.0)	104 (19.7)	123 (10.5)
MSSA	137 (22.0)	35 (06.8)	172 (14.7)
*E. coli*	47 (07.7)	51 (09.8)	98 (08.4)
*Citrobacter* sp.	34 (05.4)	–	34 (02.9)
*Klebsiella* sp.	47 (07.7)	45 (8.7)	92 (07.9)
*Proteus* sp.	19 (03.0)	41 (7.7)	60 (05.1)
*P. aeruginosa*	188 (30.1)	183 (34.3)	371 (31.8)
*Aspergillus* sp.	33 (05.3)	30 (05.8)	63 (05.4)
*Candida* sp.	22 (03.5)	46 (08.8)	68 (05.8)

CONS, coagulase negative *Staphylococcus*; MRSA, methicillin resistant *S. aureus*; MSSA, methicillin sensitive *S. aureus;* OPD, outpatients department; percent values are in parenthesis; *n* or total colonies = 1164, from the total 1230 samples; the rest 66 samples had no growth; there were 121 (63 + 68) fungal isolates.

**Figure 1:**
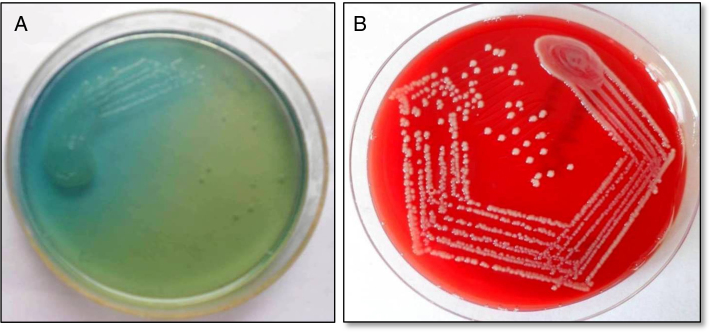
(A) Confluent growth and isolated green colonies of *P. aeruginosa* on nutrient agar plate; and (B) methicillin resistant colorless colonies of *S. aureus* (MRSA) on blood agar plate.

**Figure 2:**
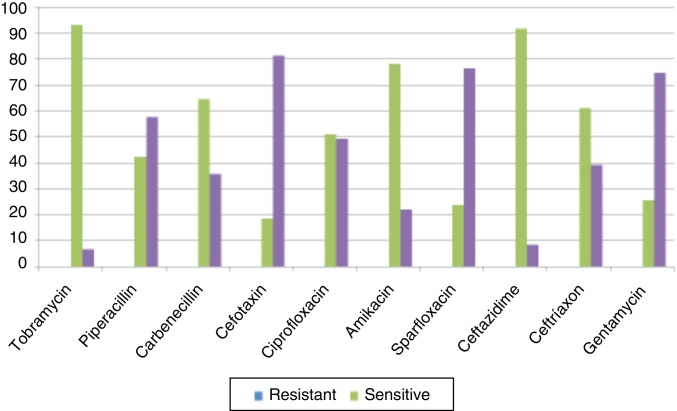
Antibiogram of *P. aeruginosa*.

**Figure 3:**
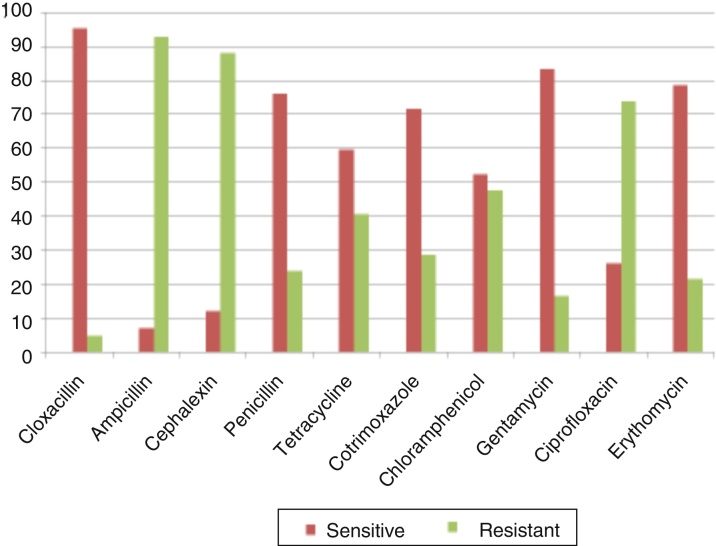
Antibiogram of *S. aureus*.

Antibiograms of the most common bacteria, *P. aeruginosa* and *S. aureus* (other than MRSA) are depicted in Fig. 1. Among *P. aeruginosa*, tobramycin 30 μg/disk had the highest susceptibility rate as 93.2%, followed by ceftazidime 30 μg/disk 91.5% and amikacin 10 μg/disk 64.4%, given in Fig. 2. And 95.2% *S. aureus* isolates were susceptible to cloxacillin 15 μg/disk, followed by 83.3% isolates to erythromycin 15 μg/disk and 78.5% isolates to gentamicin 30 μg/disk, given in Fig. 3. All MRSA isolates were MDR; however, none of those isolates were resistant to vancomycin 30 μg/disk.

Of samples of 1164 patients, 73 had complicated and 1091 patients had uncomplicated CSOM, as detailed: only 48 cases had as single bacterium isolated as a single colony, while the remaining 198 cases had two or more bacteria isolates as mixed colonies, given in Table 2. Of the total 1230 patients, 49 had post aural abscess, 12 patients had intracranial complications, 9 patients presented with facial palsy and 3 patient presented with labyrinthitis, given in Table 3. Furthermore, it was seen that the trend of intracranial complications was gradually decreasing while intracranial complications were in an increasing trend, although there was no significant change in overall incidences of CSOM. From 359 *S. aureus* samples, a total of 123 MRSA strainsand 236 strains of ‘CONS + (MSSA)’ (methicillin sensitive *S. aureus*) strains, as both single and mixed colonies. The minimum inhibitory concentration (MIC) range against oxacillin was 16–512 μg/mL, the MIC range was 1–4 μg/mL, for MRSA and ‘CONS + MSSA’. These MIC values confirmed the presence of MRSA strains, as the break point for being resistant to oxacillin was ≥4 μg/mL, given in Table 4.

**Table 2:** Numbers of growing organisms from cultures of ear discharge samples in patients with complicated and uncomplicated CSOM.

Types of organisms	Complicated CSOM	Uncomplicated CSOM	Total
As single colony	48 (0.05)	581 (0.49)	*n*_1_ = 629 (0.54)
As mixed colonies	25 (0.02)	510 (0.43)	*n*_2_ = 535 (0.45)
Total	73 (0.06)	1091 (0.94)	*n* = 1164 (100)

See note of Table 1.

**Table 3:** Numbers of patients with complications as comorbidities causing CSOM in 3 years.

Year	Facial palsy	Intracranial complication	Post-aural abscess	Labyrinthitis	Total
2012	3	3	16	2	24
2013	2	4	18	0	24
2014	4	5	15	1	25
Total	9 (12.9)	12 (16.6)	49 (67.1)	3 (3.3)	73 (100)

Percent values are in parenthesis.

**Table 4:** Detection of MRSA and ‘CONS + MSSA’ isolates based on MIC values due to oxacillin in a 12 × 8 micro-titer plate.

Well	Oxacillin (μg/mL)	Number of isolates	
		MRSA = 123	CONS + MSSA = 236
1	0	123	236
2	≤0.25	–	–
3	0.5	–	–
4	1	–	83
5	2	–	75
6	4	–	78
7	8	–	–
8	16	23	–
9	32	26	–
10	64	27	–
11	128	29	–
12	≥256	28	–

The oxacillin stock solution of 512 μg/mL was serially diluted at each successive well, from the 12th well for final concentration of 0.25 μg/mL oxacillin at the 2nd well; –, no growth; total *Staphylococcus* sp. = MRSA with 123 + (CONS + MSSA) with 236 = 359 colonies. Results of the second repeated experiment are presented.

## Discussion

CSOM is a disease associated with the structural change in middle ear; and permanent abnormality of pars tensa or pars flaccid, mostly occur as sequelae of long standing middle ear effusion, inadequately treated AOM, eustachian tube dysfunction or even from a negative middle ear pressure. In the developing countries, poverty, ignorance, dearth of specialists and limited access to medical care amongst others conspire to worsen the occurrence and complications of CSOM^17^; poor living conditions, poor access to medical care, inadequate medical treatment, recurrent upper respiratory tract infections and nasal diseases have been recognized as risk factors for CSOM.^18^ Atticoantral disease most commonly is involved with the pars flaccida and posterior superior quadrant of pars tensa. It is characterized by the formation of a retraction pocket in which, keratin and desquamated epithelial debris accumulate to produce cholesteatoma; eventually it is considered to be a dangerous form of the disease because of the development of several intracranial and extracranial complications.^18^ Moreover, staphylococci are a part of the normal flora, but those remain invasive causing a variety of body infections. *S. aureus* is the most notorious nosocomial pathogen and in community too.^11^

Although the clinical relevance of CONS is still controversial, patients at risk of CONS infections include neonates, those with intravascular catheters, prosthetic devices and surgical wounds in immune-compromised individuals. The remarkable ability of *S. aureus* and CONS to acquire antibiotic resistance limits therapeutic options, attended with high rates of morbidity and mortality, including costs of hospitalization.^19^ Particularly, several clonal variants of *S. aureus* and MRSA were reported resistant to the penicillin group of antibiotics, methicillin/oxacillin. Moreover, in a German study, it was reported that a majority of MRSA strains were from wound infections (56.9%), with pneumonia cases being the second most common (21.0%), followed by BSI (15.1%).^11^

## Conclusion

MDR strains of *P. aeruginosa* and MRSA were most prevalent is ear discharges of patients with CSOM. Of the total 1164, 49 patients presented post aural abscess, 12 patients had intracranial complications, 9 patients had facial palsy and 3 patients had labyrinthitis. This study revealed ciprofloxacin as less effective in the treatment of active CSOM, and tobramycin and cloxacillin could preferably be used to treat CSOM.

## Funding

This work was supported by the major research project n° BT/PR8214/PBD/17/863/2013 on bacterial infections, from Department of Biotechnology (DBT), Govt. of India, New Delhi, awarded to RN Padhy. This work is a part of PhD thesis of SN Rath, a JRF in the DBT project, in Biotechnology of S‘O’A University, Bhubaneswar. We are grateful to Prof. Rankanidhi Samal, for critical appreciation and thankful to Prof. Gangadhara Sahoo, Dean, IMS and Sum Hospital, Bhubaneswar, for extended facilities.

## Conflicts of interest

The authors declare no conflicts of interest.
